# Enhancing diagnostic reliability in non-invasive health Monitoring: An analytical framework for optimizing magnetic sensor-skin interactions in biomedical applications

**DOI:** 10.1016/j.mtbio.2025.102259

**Published:** 2025-09-02

**Authors:** Wasim Ullah Khan, Mohammed Alissa, Huawei Ma, Uzair Aslam Bhatti, Abdullah Alghamdi, Mohammed A. Alshehri, Abdullah Albelasi

**Affiliations:** a School of Information Engineering, Yango University, Fuzhou, 350015, Fujian, China; b Department of Medical Laboratory, College of Applied Medical Sciences, Prince Sattam bin Abdulaziz University, Al-Kharj, 11942, Saudi Arabia; c School of Information Engineering, Hangzhou Medical College, No. 8, Yikang Street, Lin'an District, Hangzhou, 311399, China; d School of Information and Communication Engineering, Hainan University, China; e Department of Medical Laboratories, College of Applied Medical Sciences, Shaqra University, Shaqra, 11961, Saudi Arabia

**Keywords:** Magnetic sensors, Optical sensors, Sensor-skin coupling, Non-invasive monitoring, Diagnostic accuracy, Biocompatible interfaces, Wearable devices, And measurement uncertainties

## Abstract

Magnetic sensors present a transformative solution for non-invasive biomedical monitoring by overcoming critical limitations associated with conventional sensing technologies, such as optical sensors, whose performance degrades due to sensor-skin coupling effects. We systematically examine the sensor-skin coupling effect, emphasizing its impact on diagnostic accuracy. Our analysis reveals the complex challenges associated with sensor-skin interfaces, including biomechanical, pigmentary, and textural variations that affect sensor performance. We introduce a novel methodology that combines advanced biomaterial development, adaptive calibration techniques, and sophisticated signal processing algorithms. Our findings highlight that skin-specific characteristics contribute to measurement uncertainties in existing sensing technologies, indicating an urgent need for adaptive interface solutions. We propose a conceptual model for developing biocompatible sensor interfaces that maintain measurement integrity across varied physiological conditions, drawing insights from materials science, bioengineering, and clinical research. This review provides a comprehensive examination of sensor-skin interactions and outlines a roadmap for next-generation health monitoring technologies, along with strategic recommendations for enhancing the reliability of non-invasive diagnostics through innovative biomaterial solutions.

## Graphical abstract

**Figure:**
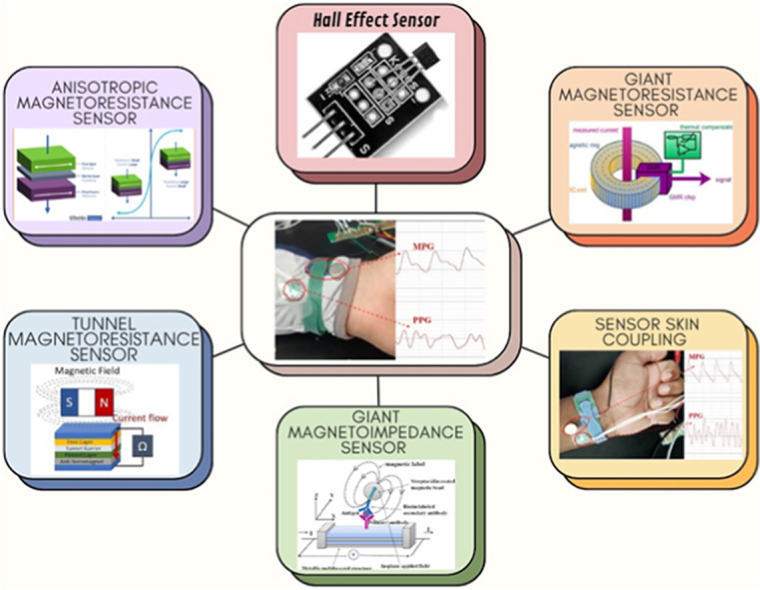

## 1 Introduction

**Fig. 1:**
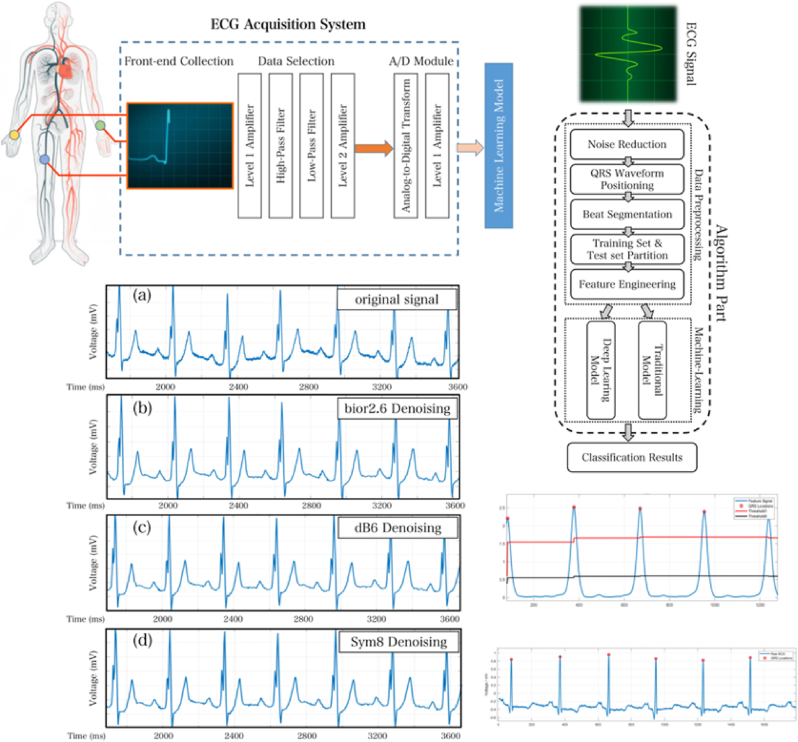
The algorithm components of the data processing and machine learning modules for the ECG acquisition system are illustrated. The wavelet denoising effects are demonstrated with (a) the original signal before denoising, (b) the denoising effect using the wavelet basis function bior2.6, (c) the denoising effect using the wavelet basis function db6, and (d) the denoising effect using the wavelet basis function sym8. QRS waveform localization results are shown, with red circles marking the peak positions of detected QRS waves, highlighting the effects of filtering and the experimental results for identifying QRS waves. Reproduced under the CC BY 4.0 license of MDPI [9]. (For interpretation of the references to color in this figure legend, the reader is referred to the Web version of this article.)

In biomedical applications, various sensor technologies play a crucial role in monitoring physiological parameters, each with its unique strengths and weaknesses [1]. Optical sensors, such as Photoplethysmography (PPG) sensors and pulse oximeters, are widely used for non-invasive monitoring [2]. PPG sensors measure blood volume changes in microvascular tissues, while pulse oximetry is a noninvasive medical technique that measures the amount of oxygen in a person's blood by shining light through their skin. It is widely used in medical care and is considered as important as the 4 traditional vital signs. They work by using two wavelengths of light to measure the absorption of oxygenated and deoxygenated hemoglobin, therefore providing critical data for patient management [3]. **Reflective optical sensors** are also significant in this context. These sensors utilize light reflection to assess physiological signals and can be integrated into wearable devices. They are particularly useful for monitoring heart rate and other vital signs in situations where direct skin contact may be impractical, such as in patients with skin conditions or during physical activity [4]. Other notable optical sensors include Near-Infrared Spectroscopy (NIRS), which measures tissue oxygenation and blood flow, and Optical Coherence Tomography (OCT), which provides high-resolution imaging for diagnostic purposes [5,6]. Fluorescence sensors are employed for detecting biomarkers in tissues, while contactless infrared thermometers are used for non-invasive temperature measurements [7,8]. Electrocardiograms (ECG) are widely used in clinical settings to monitor heart activity by recording electrical signals generated by the heart. ECGs provide critical information for diagnosing arrhythmias, myocardial infarctions, and other cardiovascular conditions. They require electrodes to be placed on the patient's skin [9]. The development of a portable ECG acquisition and analysis system is built upon machine learning and consists of two main components: data preprocessing and machine learning models for ECG signal classification shown in Fig. 1. Despite their various advantages, these optical sensors and ECG face limitations, primarily related to their dependency on light transmission and sensor-skin interaction, which can affect accuracy and reliability [10] (see Fig. 11).

Magnetic sensors present a compelling alternative, leveraging the unique properties of magnetic fields to detect physiological changes. These sensors can penetrate barriers such as clothing without loss of signal integrity, making them particularly suitable for accurate physiological measurements. They operate based on changes in magnetic fields caused by physiological processes, such as variations in blood volume [11]. Examples of devices include Magnetic Cardiogram (MCG), magnetic resonance imaging (MRI) sensors, and magnetic induction sensors for monitoring blood flow, which facilitate non-invasive blood volume measurement and monitoring of circulatory conditions [12]. Recent advancements in magnetic sensor technology focus on miniaturization and integration with other sensing modalities, allowing for the development of multi-functional devices that provide comprehensive health monitoring solutions [13]. Magnetic sensors enhance patient monitoring and diagnosis [14,15]. These sensors detect variations in magnetic fields and convert them into electrical impulses to assess various physiological indicators [16]. Wearable technologies and advanced diagnostic devices utilize magnetic sensors, demonstrating their versatility. The applications of different magnetic sensors in diverse circumstances fluctuate [17]. Magnetic sensor-based methods offer several advantages over other non-invasive monitoring techniques, making them a valuable tool in various applications. One primary benefit is their high sensitivity to magnetic fields, which enables precise measurements of physiological parameters, such as heart rate and motion, without direct contact with the body. This non-invasive approach minimizes patient discomfort and allows for continuous monitoring in real-time, which is particularly advantageous in clinical settings and wearable technologies. Additionally, magnetic sensors are typically robust and can operate effectively in diverse environmental conditions, making them suitable for both indoor and outdoor applications [18]. Their compact size and light weight also facilitate easy integration into portable devices, enhancing user convenience. Furthermore, magnetic sensor technologies exhibit low power consumption, which is critical for battery-operated devices [19].

**Fig. 2:**
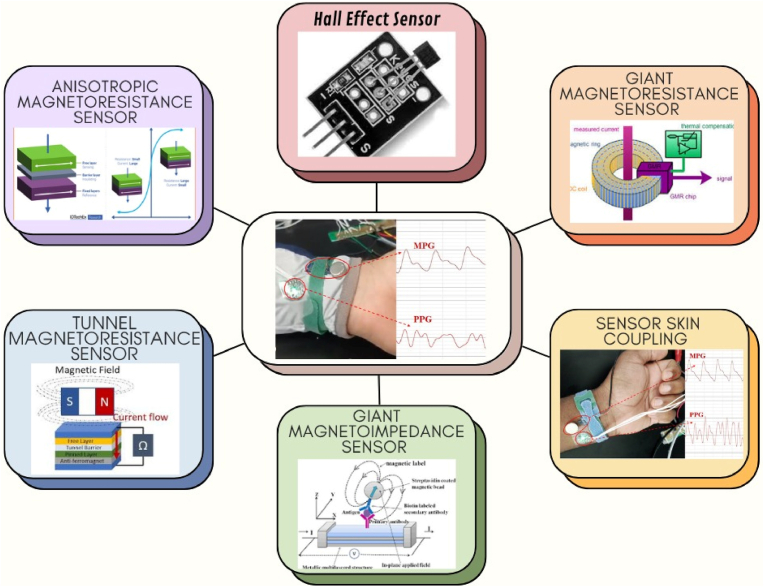
A graphical representation of various magnetic sensors, including HES, AMR, GMR, TMR, and GMI sensors.

Overall, these advantages position magnetic sensor-based methods as a leading choice for innovative monitoring solutions in healthcare and beyond. It is common to encounter Hall Effect Sensors (HESs), Anisotropic Magnetoresistance (AMRs), Giant Magnetoresistance (GMR), Tunnel Magnetoresistance (TMR), and Giant Magnetoimpedance (GMI) [[20], [21], [22], [23], [24], [25], [26], [27], [28]]. Each has unique advantages; for example, HESs are optimal for fundamental monitoring due to their simplicity and durability, while AMR and GMI are highly sensitive to weaker magnetic fields. The proposed study presents numerous types of magnetic sensors and their applications in the biomedical field, as illustrated in Fig. 2.

Despite their advantages, challenges persist, such as the sensor skin coupling effect, where skin features influence sensor readings [[29], [30], [31], [32], [33], [34], [35], [36], [37]]. Skin's interaction with the sensor's magnetic field can vary due to skin thickness, moisture, and pigmentation, affecting diagnostic and therapeutic applications [[38], [39], [40]]. Accurate healthcare outcomes are hindered by skin coupling, impacting continuous assessment of indicators like heart rate (HR) and blood oxygen levels (BOL) [20,41]. Variability in data due to skin tone and sensor-skin coupling can lead to misdiagnosis and decreased patient confidence, especially in marginalized populations [42,43]. Recent improvements in wearable health devices target these constraints, addressing issues like motion artifacts and material interferences [[44], [45], [46], [47]]. Fingertip devices using magnetic sensors for pulse detection have shown reliability even under challenging conditions, like liquid immersion [48]. Advancements in GMR sensors for HR monitoring have demonstrated precision in identifying magneto-plethysmography (MPG) signals, establishing credibility as alternatives to traditional HR monitors [49]. TMR sensors have improved cardiac diagnostics by recognizing essential cardiac signals without averaging, providing cost-effective non-invasive cardiac monitoring [50]. Physiological signals obtained from sensors require preprocessing to accurately extract essential features, thereby improving the device's accuracy. To enhance system performance, it is necessary to apply noise reduction techniques [51,52].

**Fig. 3:**
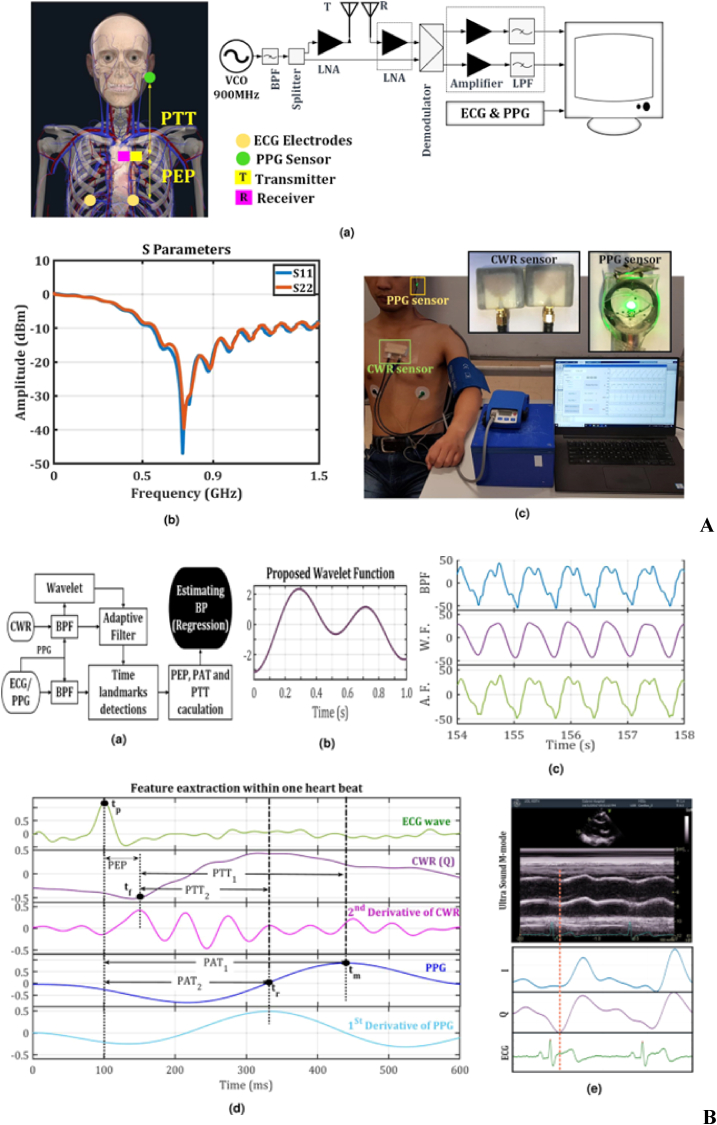

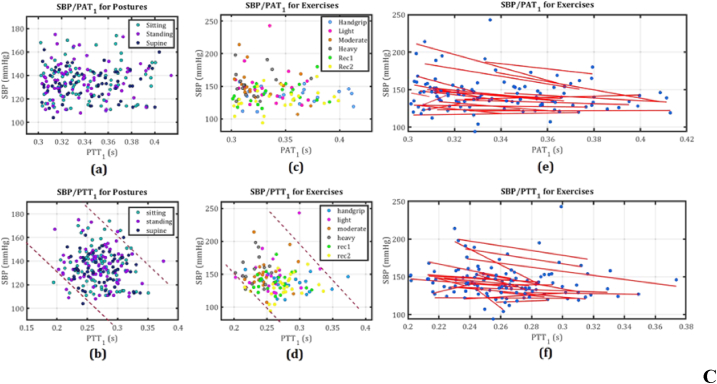
The continuous wave radar (CWR) system setup and sensor placement are shown in **A**, including (a) the system setup and sensor placement, (b) S-parameters of CWR antennas, and (c) sensor placement for CWR, ECG, and PPG on an exercise bike. **B** presents (a) a block diagram for blood pressure estimation, (b) proposed wavelet function design, (c) processed CWR signal sample for Subject 1, (d) ECG, CWR, PPG, and their derivatives for Subject 1 during blood pressure estimation, and (e) a comparison of CWR signal and ultrasound M-mode signal. C includes (a) SBPs/PAT1 for three posture tasks, (b) SBPs/PTT1 for three posture tasks, (c, d) SBPs/PAT1 for six exercise tasks, and (e, f) SBPs and PTT1 for six exercise tasks with fitted curves for each subject. Reproduced under the CC BY 4.0 license of Springer Nature [56].

A summary and comparison of magnetic materials and sensors is provided, detailing their types, properties, and applications. This includes ferromagnetic metals such as iron, cobalt, and nickel, rare-earth magnets like samarium cobalt and neodymium, ceramic ferrites, and various sensors such as HESs, AMRs, GMRs, TMRs, and GMIs. Their roles in modern technology are highlighted in Table 1. Enhancing sensor materials and adaptive calibration procedures aim to improve reading consistency across various skin types, fostering equitable healthcare solutions [53,54]. This study examines the sensor skin coupling effect's impact on magnetic sensors in healthcare, exploring mechanisms, skin's physical qualities, magnetic field interaction, and innovative enhancement methods, focusing on sensor technology, signal processing, and testing methodologies [55]. It identifies research deficiencies and proposes future endeavors to enhance magnetic sensor technology for universal access to dependable health monitoring, mitigating the skin coupling effect to benefit various individuals. The use of CWR, PPG, and ECG sensors for continuous SBP measurements are shown in Fig. 3 [56].

**Table 1:** Summary and comparison of Currently used magnetic materials and magnetic sensors, highlighting their properties and applications.

Material/Sensor	Type	Magnetic Properties	Applications
Iron (Fe)	Ferromagnetic	High permeability, high saturation magnetization	Transformers, electromagnets, magnetic storage
Cobalt (Co)	Ferromagnetic	High coercivity, good thermal stability	Hard magnets, magnetic recording media
Nickel (Ni)	Ferromagnetic	Good ductility, moderate permeability	Battery electrodes, magnetic shielding
Neodymium (NdFeB)	Rare-earth magnet	Very high magnetic strength	High-performance motors, magnetic resonance imaging
Samarium Cobalt (SmCo)	Rare-earth magnet	High temperature stability, corrosion-resistant	Aerospace, military applications
Alnico	Alloy (Aluminum, Nickel, Cobalt)	Good thermal stability, moderate strength	Electric guitar pickups, sensors
Ferrites	Ceramic magnetic material	Low electrical conductivity, good high-frequency performance	Microwave devices, inductors, transformers
Hall Effect Sensors (HESs)	Sensor	Voltage output proportional to magnetic field	Position sensing, current measurement
Anisotropic Magnetoresistance (AMRs)	Sensor	Change in resistance with magnetization direction	Magnetic field sensing, reading heads in hard drives
Giant Magnetoresistance (GMR)	Composite material	Change in resistance in magnetic fields	Data storage devices, sensors
Tunnel Magnetoresistance (TMR)	Composite material	Very high sensitivity to magnetic fields	Spintronic devices, high-density data storage
Giant Magnetoimpedance (GMI)	Sensor	Large change in impedance with magnetic field	Magnetic field sensing, biosensing applications

The highlight of study contributes the following insights.

• Addresses the need for non-invasive magnetic sensor techniques for ongoing health surveillance and early detection.
• Investigates the impact of sensor skin coupling on the efficacy of magnetic sensors, resulting in varying diagnostic precision and health surveillance.
• Examines the applications of magnetic sensors in imaging, diagnostics, and devices, emphasizing their relevance to the early detection of diseases.
• Enhances sensor consistency through adaptive calibration, signal processing, and improved materials while innovative techniques reduce sensor skin coupling.
• Identifies research voids for developing magnetic sensor-based technologies in biomedical healthcare applications to facilitate equitable health evaluations for all individuals.

## 2 Definition and mechanism of sensor-skin coupling

In physiological monitoring, the contacts between human skin and magnetic sensors are referred to as sensor-skin coupling. This dynamic is essential for accurate monitoring of vital signs such as blood oxygen saturation and heart rate. There is a possibility that the efficiency of sensors is influenced by the wetness, color, and texture of the skin [57]. The stratum corneum, the dermis, and the subcutaneous tissue each exhibit various electromagnetic and physical feature that are distinct from one another. Because of differences in magnetic permeability and conductivity, melanin has an effect on the way the skin reacts to magnetic fields, which in turn has an effect on the performance of sensors [58]. The continual and non-invasive collection of physiological data has been made possible by recent advancements in wearable sensors, which have revolutionized the process of health monitoring significantly. These devices, which make use of a wide variety of sensing technologies, have a significant potential for providing rapid health insights and individualized treatment recommendations. According to the research that has been conducted [[59], [60], [61]], wearable sensors are gradually becoming more prevalent in everyday life. This has led to an improvement in the monitoring of situations such as heart rate, glucose levels, and other essential indicators. Studies have shown that differences in the composition of the skin may influence the results of magnetic sensors [62]. Both magnetoencephalography (MEG) and MCG require precise measurements of the magnetic fields that are generated by the heart or the brain while performing the examinations. Inconsistencies in the coupling between the sensor and the skin can lead to incorrect diagnoses and inadequate care for the patient [63,64]. Because various groups are embracing new technologies, equitable healthcare is necessary. The interactions between sensor technology and skin have the potential to provide inaccurate results for some demographic groups, which can lead to an increase in health inequities and a decrease in confidence in the quality of healthcare technology [65]. The incorporation of sensor-skin technology is necessary for the provision of trustworthy health evaluations for all individuals, hence improving faith in the advancements that have been made in contemporary medicine [66,67].

## 3 Magnetic sensors applications in biomedical healthcare with a focus on sensor-skin coupling

In this section, we explore the pivotal role of magnetic sensors in biomedical healthcare and focus on a detailed examination of each specific type of magnetic sensors. Magnetic sensors are increasingly utilized for measuring vital signs through devices like smartwatches and fitness monitors. Lee et al. [68] propose a novel MPG sensor that detects changes in blood volume using time-varying magnetic fields. This sensor measures impedance changes in an exciting coil, which correlate with blood volume variations in a specific area. To validate their method, the researchers simultaneously recorded PPG and MPG signals from the index and middle fingers. The results revealed strong correlations between the sensor's signals and ultrasound Doppler blood flow velocity recordings (r = 0.9355, p < 0.01), as well as between RR intervals from the electrocardiogram and MPG signals (r = 0.9823, p < 0.01). These findings demonstrate the sensor's feasibility for blood volume monitoring. Ravanelli et al. [69] assess the Bangle. js2, a consumer-grade wrist-based wearable device, for tracking step counts and heart rate. Their study, which involved 47 participants, utilized a custom open-source application to capture data during both lab-based treadmill tests and a 24-h free-living period. While the Bangle. js2 showed systematic undercounting of steps at slower walking speeds, it achieved acceptable accuracy at 5 km/h. The device demonstrated strong agreement with the Fitbit Charge 5 for step counting (CCC = 0.90) and total steps (CCC = 0.96), and with the Polar H10 for heart rate (CCC = 0.78). Overall, the findings indicate that the Bangle. js2 is a valid open-source solution for monitoring physical activity and heart rate in real-world conditions. This summary outlines the corresponding magnetic sensing technologies and essential biomarkers relevant to the detection of cutaneous and cardiovascular disorders. Its objective is to enhance understanding of the importance of target biomarkers in both domains, as detailed in Table 2.

**Table 2:** Summary of biomarkers and magnetic sensing technologies for skin detection and heart diseases.

Biomarker	Description	Detection Technology	Notes
**Blood Volume Variations**	Fluctuations in blood volume that can indicate cardiovascular risk	GMR, TMR	Useful for predicting cardiovascular diseases
**ECG (Electrocardiogram)**	Measures electrical activity of the heart	HESs, GMR, TMR	Essential for diagnosing arrhythmias and other heart conditions
**Melanin**	Pigment responsible for skin color	HESs, GMR, TMR	Can indicate skin health
**Cortisol**	Stress hormones present in sweat	AMRs, TMR	Potential indicator of stress
**Glucose**	Sugar level in blood and interstitial fluid	GMI, GMR	Important for diabetes monitoring
**Lactate**	Indicator of metabolic activity	TMR, AMRs	Useful in athletic performance
**pH Levels**	Acidity/alkalinity of skin	HESs	Can indicate skin condition
**Uric Acid**	Associated with gout and kidney function	GMR, TMR	Indicates metabolic disorders
**Pro-inflammatory Cytokines**	Indicators of inflammation in the skin	AMRs, GMI	Important for skin diseases
**Troponin**	Protein released during heart muscle injury	TMR, GMR	Key biomarker for heart diseases
**B-type Natriuretic Peptide (BNP)**	Hormone produced by the heart in response to stress	HESs, AMRs	Indicates heart failure risk

Health monitoring devices may provide inaccurate results for individuals with varying pigmentation if they are calibrated for a specific skin tone, which could compromise their accuracy. Magnetic sensors are indispensable in clinical settings for technologies like MCG and MEG, which assess cerebral and cardiac function [70]. Xiao et al. [71] present a movable MCG system that records magnetic field signals generated by the heart's electrical activity in unshielded environments. Traditional MCG requires participants to remain still in magnetically shielded rooms, limiting its application for exercise tests and long-term monitoring. The new system, utilizing optically pumped magnetometers with a sensitivity of 140 fT/Hz^1/2, successfully captures both resting and exercise MCG signals from freely moving participants. This advancement aims to enhance the practicality of MCG in diagnosing heart diseases by reducing participant restrictions and providing greater insights into cardiac health. Yang et al. [72] develop a wearable multichannel human MCG system utilizing a spin exchange relaxation-free regime (SERF) magnetometer array. This system comprises a magnetically shielded device, a wearable SERF magnetometer array, and a computer for data acquisition and processing. The researchers successfully recorded multichannel MCG signals from a healthy subject simultaneously. To enhance data quality, independent component analysis (ICA) and empirical mode decomposition (EMD) were employed for denoising. MCG imaging was achieved to visualize the magnetic and current distribution around the heart. Validation of the MCG signals was performed by comparing them with ECG signals recorded at the same site, revealing similar waveform features and intervals. The experiments demonstrate that the wearable MCG system is reliable for detecting cardiac electromagnetic activity and provides valuable imaging capabilities.

Inconsistencies in the integration of sensors and skins may result in errors, which can compromise the accuracy of diagnostics [70]. It is imperative to rectify these discrepancies to ensure that clinical measurements are conducted with precision and speed [73]. Magnetic sensors are indispensable components of imaging systems, including MRI [74]. Mojtaba Safari et al. [75] review the use of deep learning (DL) techniques for improving MRI reconstruction, addressing challenges such as long acquisition times and motion artifacts. They discuss various DL methods, including end-to-end approaches, unrolled optimization, and federated learning, highlighting their advantages in enhancing reconstruction speed and accuracy. The review summarizes key trends, quantitative metrics, and datasets in DL-based MRI reconstruction, while emphasizing its potential to advance medical imaging. Additionally, a GitHub repository is provided to facilitate further research in this area. The MRI apparatus produces intense magnetic fields that enable the precise visualization of internal structures. The diagnostic accuracy and image quality of a patient can be influenced by the interaction between the patient's dermis and these domains [[76], [77], [78]]. Researchers are investigating strategies to improve the efficacy of sensors in a variety of epidermis types [[79], [80], [81], [82], [83], [84], [85], [86]]. Having provided an in-depth discussion of the applications of magnetic sensors, now focus on a detailed examination of each specific type of magnetic sensor.

### 3.1 Hall Effect Sensors

HESs are crucial in biomedical healthcare for accurate magnetic field measurements [87]. The Edwin Herbert Hall discovered in 1879, the Hall effect involves the interaction between electric current and magnetic fields, producing a measurable Hall voltage [88,89]. This voltage is perpendicular to both the magnetic field and current. This phenomenon occurs when a magnetic field encloses a conductor as charge carriers traverse it.

Chheng et al. [90] developed a wireless, low-power gait monitoring system using HES and magnets mounted on opposing legs to track subtle gait variations. The system measures stride width, cadence, and their variability during normal, abnormal, and irregular walking patterns. By analyzing leg gap changes via magnetic field fluctuations, it achieved 81 % accuracy in detecting abnormal strides and 100 % cadence-based identification of irregular gait revealing nuances invisible to the naked eye. This non-contact HES approach enables natural-setting gait analysis, offering potential for early disease detection (e.g., Parkinson's) and rehabilitation progress tracking without restrictive wearables. Carmo et al. [91]. Developed a wearable HES system to monitor breathing during pulmonary rehabilitation exercises. The sensor, paired with a permanent magnet, detects chest movements and compares its performance against a gold-standard airflow transducer and a piezoelectric sensor. In tests with 16 healthy participants performing 15 different exercises, the HES-based system demonstrated superior accuracy in detecting breathing patterns, with high precision (0.97) and recall (0.95), outperforming the piezoelectric sensor. It accurately measured breath cycle, inspiration, and expiration times with mean errors of ∼4 % and ∼8 %, respectively, showing no significant bias in Bland-Altman analysis. However, its performance slightly declined in exercises involving side torso stretches due to magnet displacement. The study confirms that HES-based wearables are viable for real-time respiratory monitoring, particularly in controlled rehabilitation settings, expanding the potential of magnetic sensing in healthcare applications. Kim et al [92]. Developed a portable planar Hall effect (PHE) biosensor platform for detecting magnetically-labeled biomolecules without requiring an external magnetic field. Their trilayer sensor (Ta/NiFe/Cu/IrMn/Ta), fabricated via photolithography and sputtering, achieved optimized sensitivity of 6 μV/(Oe∙mA). The system utilized self-field detection by operating in second harmonic AC mode (2f mode), eliminating the need for bulky external magnets. This approach was validated using a β-amyloid biomarker sandwich assay, demonstrating high signal-to-noise ratio (SNR) for magnetic label detection. Key advantages include miniaturization potential, improved thermal stability, and suitability for point-of-care testing, as the self-generated magnetic field enabled compact, portable diagnostic applications. The study highlights the PHE sensor's promise for label-based biomedical detection with simplified hardware requirements. The CMOS Hall-effect sensor array developed by Skucha et al. [93]. holds significant promise for biomedical applications, particularly in high-sensitivity diagnostic assays and single-cell analysis. By enabling label-free detection and precise spatial mapping of magnetic microbeads, this technology could revolutionize point-of-care testing (POCT) for example, in detecting disease biomarkers (e.g., proteins, nucleic acids) bound to functionalized beads in miniaturized lab-on-chip devices. Additionally, its ability to image bead distributions at microscale resolution supports applications like circulating tumor cell (10.13039/100007404CTC) isolation or immunomagnetic cell sorting, where tracking bead-bound cells is critical. The system's CMOS compatibility further facilitates integration into portable, low-cost platforms for rapid, automated diagnostics in resource-limited settings. Son et al. [94]. Developed a non-invasive arterial pulse monitoring system using a HES to detect blood flow dynamics at the traditional "Guan' pulse position. Their clamping pulsimeter design employs a small permanent magnet placed near the radial artery, with the HES capturing magnet movements induced by arterial pulsations eliminating the need for uncomfortable pressure cuffs. The system precisely measures key pulse waveform features (systolic, reflective, and notch peaks) and calculates heart rate/blood pressure with clinical reliability. This HES-based approach demonstrates potential for continuous cardiovascular monitoring and Traditional Chinese Medicine diagnostics, offering a comfortable, cuffless alternative to conventional tonometry. Kang et al. [95]. Investigated the effects of low-frequency acupuncture electrical stimulation (AES) on cardiovascular parameters using a HES-based clip-type pulsimeter. Over seven months, they measured SBP/DBP, heart rate (HR), and systolic time (S.time) in 80 datasets. The pulsimeter equipped with a permanent magnet and HES non-invasively tracked radial artery pulse waves. Results showed AES (30 Hz) significantly stabilized cardiovascular function: SBP decreased by 5.7 mmHg, DBP by 2.1 mmHg, and HR by 1.4 bpm, while S. time increased by 2.6 ms, indicating improved blood flow. The study demonstrates that low-frequency AES enhances hemodynamic stability and validates the HES pulsimeter as a precise tool for non-invasive cardiovascular monitoring. The Lorentz force causes charge accumulation at the edges of the conductor due to velocity oscillations of the charge carriers [96]. The sensitivity of HESs depends on the mobility of charge carriers. HESs based on Silicon are common with variable sensitivity, whereas materials like gallium arsenide (GaAs) and graphene improved performance [97,98]. HESs address integration challenges with the skin, crucial for the prediction of vital signs like blood circulation and heart rate. They are essential for fitness trackers and smartwatches, providing reliable measurements across diverse skin types through adaptive calibration and advanced signal processing [99].

**Fig. 4:**
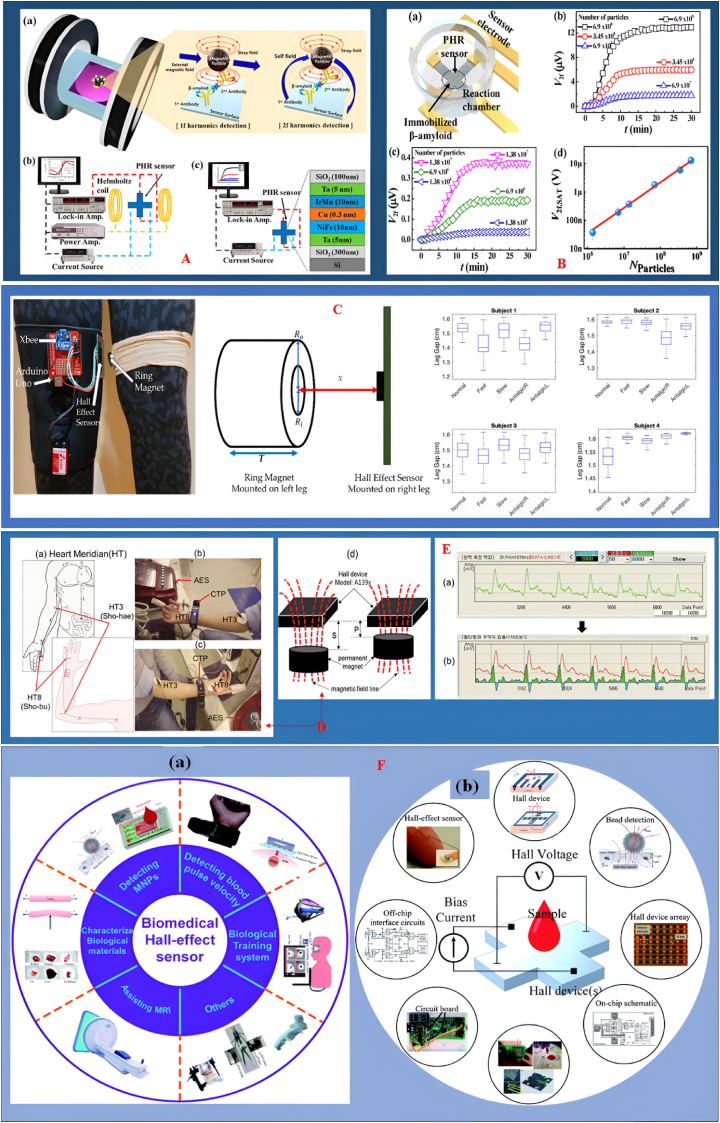
**A.** The planar Hall resistance (PHR) biosensor operates effectively in both first harmonic (1f) and second harmonic (2f) detection modes, illustrated through schematic representations and experimental setups. **B.** The PHR sensor demonstrates capability in particle detection, with time-dependent measurements of the second harmonics voltage V_2f_ indicating a linear relationship with varying particle quantities, highlighting its sensitivity (Ref [92], reproduced under the CC BY 4.0 license from MDPI). **C.** The wearable gait monitoring system prototype reveals significant leg gap variability across four subjects, underscoring its practical applications in gait analysis (Ref [90], reproduced under the CC BY 4.0 license from MDPI). **D.** The schematic of the Heart Meridian outlines key acupoints, complemented by measurement setups that illustrate the effects of acupuncture electrical stimulation (AES) on cardiovascular data, **E.** including raw and filtered results (Ref [95], reproduced under the CC BY 4.0 license from the Journal of the Korean Magnetics Society). **F** Presents the principle of HESs in biological situations. (Ref [104], reproduced under the CC BY 4.0 license of Royal Society of Chemistry).

This adaptability promotes equitable healthcare by reducing misdiagnosis risks. Recent advancements in lab-on-chip technologies and microfluidics have led to compact HESs for biosensing, detecting magnetic nanoparticles for accurate diagnosis across all skin types [100]. Flexible health monitoring systems on variable substrates enhance comprehensive health assessments by conforming to skin morphology [101]. Research shows that Hall sensors which are flexible maintain sensitivity and precision despite frequent bending, making them suitable for long-term health tracking [102,103]. A comprehensive overview of the capabilities and applications of the planar Hall resistance (PHR) biosensor is shown in Fig. 4. Part A illustrates the sensor's effectiveness in both first harmonic (1f) and second harmonic (2f) detection modes, supported by schematic representations and experimental setups. Part B demonstrates the PHR sensor's sensitivity in particle detection, showcasing time-dependent measurements of the second harmonic voltage (V2f) that reveal a linear relationship with varying particle quantities. Moreover, Part C features a prototype of the wearable gait monitoring system, highlighting significant leg gap variability across four subjects, which underscores its practical applications in gait analysis. In Part D, we outline the schematic of the Heart Meridian, detailing key acupoints and measurement setups that illustrate the effects of acupuncture electrical stimulation (AES) on cardiovascular data. This is further complemented by raw and filtered results presented in Part E. Lastly, Part F discusses the principle of Hall Effect Sensors (HESs) in biological contexts, providing a broader understanding of their implications in medical applications as discussed in (Ref 90,92,95 and 104). Addressing sensor-skin coupling is essential for diagnostic accuracy, as variations in skin can influence sensor performance. Adaptive methodologies further enhance HES reliability in clinical environments, where precise readings are vital for patient care. The versatility of HESs enables them to enhance diagnostic accuracy and improve monitoring capabilities in medical settings.

Hybrid health monitoring systems to wearable technology enhance individual health outcomes and encourage understanding of health trends [105]. Health Equity Solutions addresses healthcare disorders by offering equitable and effective health monitoring technologies, ensuring precision and reliability across all skin types [106]. Advancements in sensors will enhance healthcare quality and motivate more individuals to monitor their health, driving a personalized medical revolution [107].

### 3.2 Anisotropic Magnetoresistance

AMR is the electrical resistance of a material influenced by the angle between the electric current and its magnetization, discovered by Lord Kelvin in 1856. The ferromagnetic metals, such as iron and nickel, increased resistance when magnetized perpendicularly to the current flow [108]. The basic principle of AMR is the scattering of conduction electrons, which is affected by the alignment of the magnetic moment. Electron scattering and resistance diminish when magnetization is perpendicular to the current, though resistance escalates when magnetization is orthogonal to the current due to dispersion [109]. Permalloy, a nickel-iron alloy, is commonly utilized in AMR sensors because of its manufacturability and sensitivity [110]. Despite AMR sensors being generally less sensitive than GMR and TMR sensors, they are favored for their manufacturing simplicity and dependability [111]. The use of AMR sensors in biomedical healthcare is vital for sensor-skin coupling, which is essential for wearable health monitoring devices [112]. Smartwatches and fitness trackers utilizing AMR technology may measure essential indications such as blood oxygen saturation and heart rate, irrespective of skin type [113]. The design flexibility of AMR sensors allows elastic devices to conform to the skin, ensuring precise measurements during repetitive tasks and preserving sensitivity under mechanical strain. AMR sensors are highly proficient in detecting magnetic nanoparticles, rendering them an optimal selection for magnetic biosensing [114]. AMR sensors can precisely detect biological markers, crucial for accurate diagnostics and equitable health assessments, by detecting the magnetic fields generated by these nanoparticles [36]. The diagnostic accuracy of clinical settings can be improved with the incorporation of AMR sensors into microfluidic systems for the real-time identification of magnetic particles [37]. AMR sensors ensure reliable performance in real-time health monitoring and diagnostics owing to their durability and versatility [115]. The possibility for a transformation in healthcare delivery arises from the continuous progress of AMR sensor technology, which enhances health monitoring capacities and diagnostic accuracy. AMR sensors mitigate healthcare inequities across various demographic groups by guaranteeing that health monitoring devices are compatible with various skins. The importance of this is highlighted by the increasing popularity of data-driven healthcare solutions and individualized therapy. The use of AMR sensors in healthcare systems can enable the collection of substantial data that reflects health trends across diverse populations, thus aiding in the formulation of more accurately targeted interventions and policies [116].

Fabiano et al. [117]. Developed a non-invasive method to monitor magnetic fields produced by markers and tracers in the digestive system. This system utilizes coils to generate an alternating current (AC) magnetic field, which is measured by AMR sensors positioned at the coil's center. This approach avoids radiation exposure and is cost-effective. Monshat et al. [118] presented a genetic analysis system to identify specific genes and their mutations, aiming to prevent antibiotic misuse and slow the development of antimicrobial resistance (AMR). Their integrated AMR sensor, which combines DNA microarrays with a temperature control device, successfully amplified and identified eight AMR-related genes from bacteria such as Klebsiella pneumoniae and Escherichia coli. Paixao et al. [119] designed an AMR sensor system for assessing gastrointestinal motility. By mounting four sensors on a susceptometer, they monitored gastric activity contractions in sedated dogs, comparing results with traditional manometry. The placement of the sensors was critical, as the system's effectiveness depended on all sensors functioning correctly. Arash et al. [120] introduced a magnetic measuring device for smart knee prosthetics, integrating AMR sensors to track knee movements. This system includes two permanent magnets within the prosthesis and an array of AMR sensors, employing various angle estimation methods to monitor flexion and extension accurately. Leopoldo et al. [121] explored the detection of magnetite nanoparticles (MN) in cancer diagnostics and therapy. Their study utilized an AMR-coupled alternating current biosusceptometry (ACB) system to assess the sensitivity of detecting MN at different concentrations and distances, although they noted it was less sensitive than conventional ACB systems. Giovanni et al. [122] detailed a real-time magnetoresistive sensor designed to detect DNA point mutations. This sensor achieves a detection limit of approximately 160 pM for DNA, utilizing a unique sensing geometry to measure the quantity of magnetized beads. This advancement is crucial for improving microfluidic devices used in DNA hybridization studies. Vera et al. [123] presents an implantable neural interface using AMR sensors made of La_0_._67_Sr_0_._33_MnO_3_ (LSMO), a ferromagnetic material engineered for high sensitivity and low noise even at body temperature (37 °C). The researchers optimized LSMO film thickness and temperature response, achieving an exceptional sensitivity of ∼400 %/T and detectivity as low as 2 nT Hz^−1^/^2^ at 1 Hz and 0.3 nT Hz^−1^/^2^ at 1 kHz, making them suitable for detecting weak magnetic signals from neural activity. The miniaturized sensors, coated with biocompatible PDMS, passed biocompatibility tests, confirming their suitability for implantable applications.

**Fig. 5:**
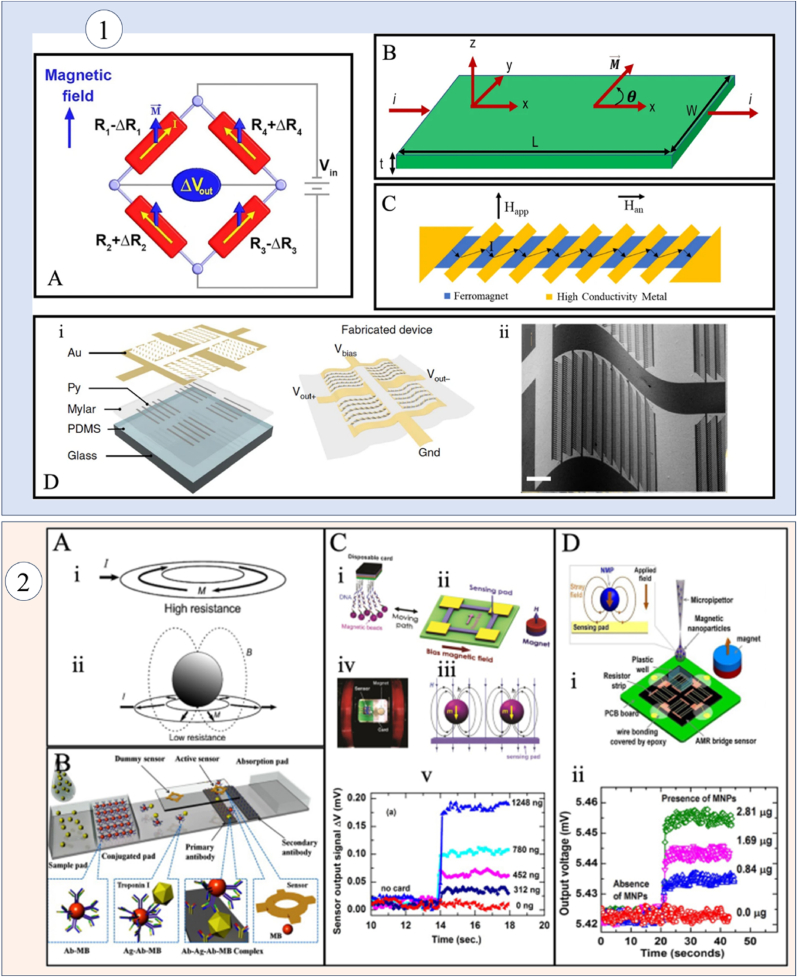
**(1)** An AMR Wheatstone bridge is illustrated, showcasing its fundamental structure (A). The diagram of a thin film AMR highlights its configuration (B). A barber-pole structure demonstrates changing current direction at an angle relative to the anisotropy field, facilitating linear magnetoresistance (C). The fabrication process for an AMR-based e-skin compass is detailed, accompanied by an SEM image of the device exhibiting a 200 μm bending radius (D). **(2)** The AMR ring resonator is depicted, showing maximum resistance, which decreases due to the presence of magnetic nanoparticles (MNPs) (A). Numerical modeling of nitrocellulose (NC) membrane detection is presented using an AMR/PHR hybrid sensor (B). An experimental setup for disposable card detection illustrates the procedure and captures recorded magnetic particle signals (C). MNP detection is achieved through a combination of six series and parallel resistor sensors, with voltage signals indicating the presence or absence of Fe_3_O_4_-chitosan MNPs (D). Reproduced under the CC BY 4.0 License of Springer Nature [116].

The diagram illustrates the AMR Wheatstone bridge and the schematic of an AMR thin film, both critical components of AMR technology depicted in Fig. 5. It showcases the barber-pole configuration, which achieves linear magnetoresistance by effectively altering the current path at an angle relative to the anisotropy field. Additionally, the schematic includes fabrication details for the AMR-based e-skin compass, accompanied by a SEM image of the device featuring a bending radius of 200 μm, highlighting its integration potential and adaptability for wearable applications. Furthermore, the figure presents a diagram of an AMR ring resonator, demonstrating how the maximum resistance state decreases to a minimum resistance in the presence of magnetic nanoparticles. This section also illustrates numerical modeling of nitrocellulose membrane detection using an AMR/PHR hybrid sensor and outlines the experimental setup for a disposable card detection system, along with the magnetic particle signals recorded by the sensor.

To conclude, AMR sensors signify an important progression in biomedical healthcare, mainly in the improvement of sensor-skin adhesion. Their capability to deliver precise and dependable measurements across various skin types is vital for the advancement of health monitoring technology that is fair and equitable. With ongoing research and technological breakthroughs, AMR sensors play an important role in providing comprehensive healthcare solutions, confirming accurate predictions of all subjects. Recent inventions and execution of AMR sensors will raise healthcare superiority and encourage inclusivity in health monitoring, finally leading to enhanced health consequences for diverse subjects.

### 3.3 Giant Magnetoresistance

A GMR sensor is a magnetic sensing sensor that identifies variations in magnetic fields by employing materials that exhibit a significant rise in electrical resistance due to the alignment of magnetic moments within a multi-layered configuration [124]. These sensors are widely utilized in diverse applications, including automobile sensors, medicinal instrumentation, data storage devices, and other magnetics-related fields. [125]. In recognition of this discovery, Albert Fert and Peter Grünberg were awarded the Nobel Prize in Physics in 2007. In GMR sensor configurations, one magnetic layer is constant, while the other, the unbound layer, undergoes magnetization changes in response to an external magnetic field [24]. GMR sensors are the preferred choice for biomedical applications due to their reduced power consumption, minimal noise, and high sensitivity. In applications that require efficient sensor-skin integration, GMR sensors have recently gained prominence [22,126]. This connectivity is indispensable for health monitoring devices that are worn on the body. Biological constituents are identified through magnetic nanoparticles, making GMR sensors indispensable in magnetic biosensing. This capability allows GMR to predict biomarkers and diseases by detecting the magnetic fields produced surrounding nanoparticles, which is beneficial in the field of clinical diagnostics. GMR sensors have the capacity to detect circulating tumor DNA (ctDNA) in samples of blood, thereby improving the early prediction of cancer [127,128]. This non-invasive method helps doctors even with slight attentions to methylated ctDNA, so helping in the implementation of timely therapies. Improving the GMR sensor-skin interface is essential for optimizing ctDNA detection and enhancing the efficacy of treatment [129]. Magnetic particles used to label cells for measurement, GMR may serve as a cost-effective alternative to fluorescence-based flow cytometry. The skin-coupling sensors developed for continuous health monitoring emphasized the potential for sensor-skin coupling in wearable devices, which is offered by flexible GMR sensor technology [130]. The adaptability of GMR sensors in biomedical and environmental disciplines is demonstrated by their ability to detect contaminants in drinking water. The design for extracting MCG is based on precise measurements, which are essential for public health compliance [131].

**Fig. 6:**
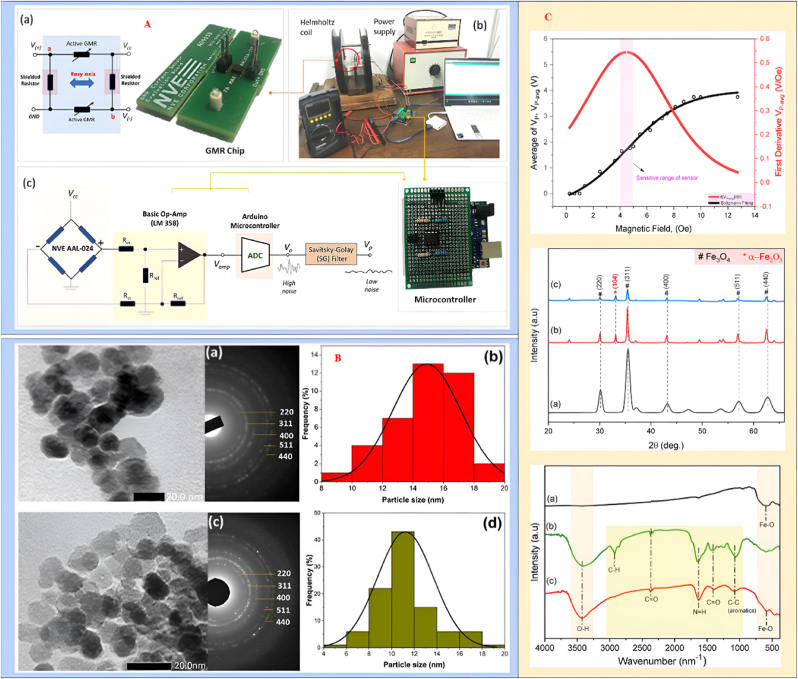
The layout and photograph of the NVE GMR chip are presented, along with the experimental setup and the schematic of the measurement configuration **(A)**. Transmission electron microscopy (TEM) and selected area electron diffraction (SAED) images depict Fe_3_O_4_ alongside size distribution images **(B)**. Additional TEM and SAED images illustrate GS-Fe_3_O_4_ at 10 mL, accompanied by corresponding size distribution images **(C)**. A graph shows the dependence of the average output voltage V_P−avg_ and its derivative on the magnetic field for the bare chip GMR sensor, highlighting sharp peaks in the crystalline profiles of Fe_3_O_4_, GS-Fe_3_O_4_ at 10 mL, and GS-Fe_3_O_4_ at 20 mL. Reproduced under the CC BY 4.0 license from the Journal of Science [130].

Wang et al. [132] developed a highly sensitive immune biosensing system utilizing a large GMR array to detect various molecular markers, including PAPP-A, PCSK9, and ST2, which are important diagnostic indicators for heart disease. This innovative system features a compact 16 mm × 16 mm chip with 64 nanoscale sensors that operate independently and simultaneously. By replacing traditional wire bonding with a lab-based probe station, this setup allows for rapid testing directly on the chip, paving the way for high-throughput screening methods in future research. Georgios et al. [133] introduced a microfluidic platform capable of automating and controlling the quantification of cancer cells isolated from whole blood samples. This system employs functionalized magnetic particles (MPs) to label and isolate cancer cells, with a GMR sensor integrated to facilitate the detection and quantification of these magnetically tagged cells. Experiments conducted with Jurkat leukemia cells demonstrated the platform's potential, although its effectiveness with whole blood samples requires further validation. Marius et al. [134] explored the interactions between magnetic particles and GMR sensors for biosensing applications. Their micromagnetic simulations indicated that the GMR sensor's response could be influenced by the arrangement of magnetic particles on its surface. By optimizing the placement of these beads and matching their size to the sensor, they were able to enhance sensitivity and achieve measurable changes in resistance, although low levels of GMR response were observed. Bhagaban et al. [135] designed a short-range magnetic pressure transducer featuring thermal compensation. This multifunctional sensor consists of a permanent magnet, a corrugated stainless-steel diaphragm, a GMR sensor, and a signal processing unit. The pressure applied by the diaphragm affects the magnetic flux, with the GMR sensor positioned asymmetrically along the magnetic axis to produce a voltage output proportional to the force per unit area. Calibration tests were conducted under varying temperature conditions to assess drift and uniformity. Shoshi et al. [136] utilized lab-on-a-chip technology to study the absorption of magnetic particles by human fibroblast (NHDF) cells in real-time. They investigated the mobility and distribution of these particles during phagocytosis using GMR sensors. Their findings indicated a cellular absorption rate under physiological conditions, with additional experiments conducted at lower temperatures to simulate inhibited phagocytic activity. Lei et al. [137] created a microfluidic model integrated with a GMR biosensor to examine the affinities and dynamic behaviors of biological molecules. This system has proven effective for real-time detection of interactions, such as between streptavidin and biotin. They also studied the adsorption of nanoparticles ranging from 50 to 500 nm, fitting the results to various adsorption models to analyze the thermal and kinetic properties of different medical compounds. The noise characteristics of the GMR sensor at 4 K are illustrated, along with a mixed GMR configuration for MCG recording as shown in Fig. 6. The experimental setup for evaluating noise levels compares the integrated sensor with a standalone GMR sensor, demonstrating their effectiveness in MCG applications. For the bare GMR sensor, the average output voltage (VP-avg) exhibited a linear increase with the magnetic field (HB) between 2 and 8 Oe, reaching peak sensitivity at a specific bias magnetic field. The transfer curve for VP-avg against HB displayed a quasi-step-like pattern, indicating optimal responsiveness in the 4 to 5 Oe range. X-ray diffraction (XRD) patterns confirmed that the structural integrity of Fe3O4 and GS-Fe3O4 (10 and 20 mL) remained largely unchanged with the addition of Moringa oleifera (MO). The average crystallite sizes were approximately 11.7 nm for Fe3O4 and 13.7 nm and 14.7 nm for the GS-Fe3O4 samples. Fourier-transform infrared (FTIR) spectra indicated the presence of hydroxyl groups, while transmission electron microscopy (TEM) images demonstrated that GS-Fe3O4 nanoparticles exhibited a more dispersive morphology and reduced aggregation, likely due to the MO coating.

In summary, GMR sensors, especially with sensor-skin technology, represent significant advancements in biomedical healthcare. Their sensitivity, adaptability, and precision make them appropriate for wearable devices to monitor and diagnose health status. As research progresses, GMR sensors are expected to enhance health assessments' accuracy. It is imperative to establish a strong connection with the epidermis to improve the functionality of wearable technologies and promote egalitarian healthcare. GMR sensors contribute to the mitigation of healthcare disparities and the provision of reliable health monitoring services for a wide range of populations by fortifying this relationship.

### 3.4 Tunnel magnetoresistance

The TMR sensors operate within magnetic tunnel junctions (MTJs), which are partitioned by a narrow insulating barrier between two ferromagnetic layers [138]. This configuration allows TMR sensors to detect fluctuations in magnetic fields with exceptional sensitivity, which is advantageous for biological applications [27].

**Fig. 7:**
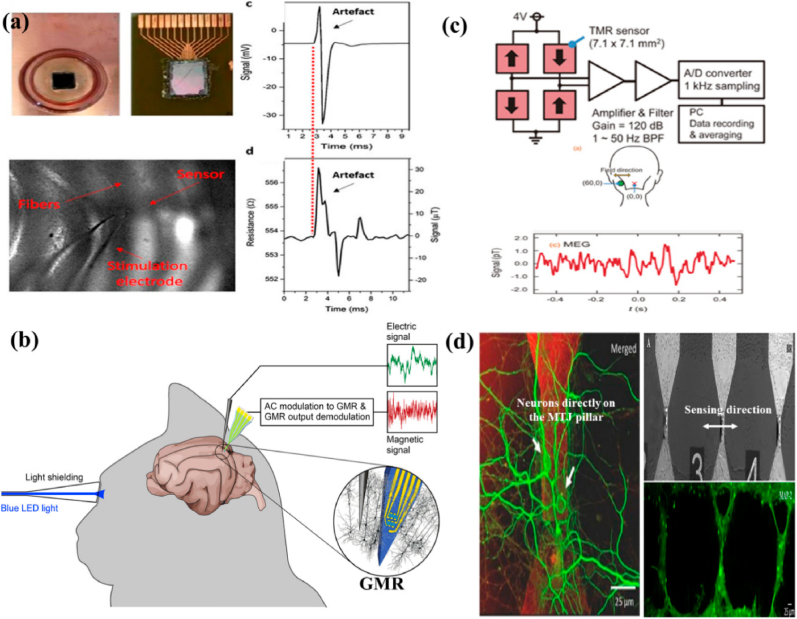
(A) An MTJ sensor array and the in vitro setup used to demonstrate neural responses. The signals, measured in millivolts (mV), and the resistance, measured in ohms (Ω), represent local field potential (LFP) recordings and the MTJ sensor's response, respectively. Both signals have similar shapes. (b) The in vivo setup for recording neural responses from the rat cerebral cortex. (c) Four TMR sensors configured in a Wheatstone Bridge arrangement, along with the neural response recorded by this sensor setup, (d) Orientation of the axons of the neurons. Reproduced under the CC BY 4.0 license from MDPI [139].

**Fig. 8:**
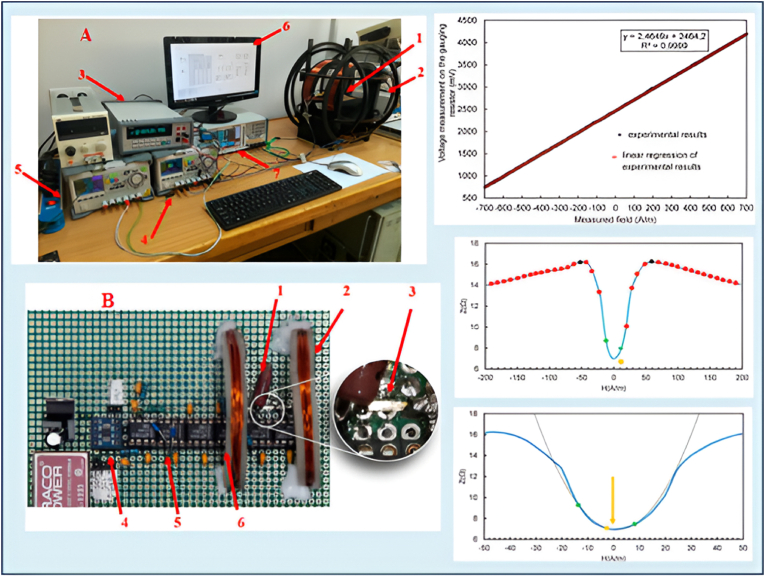
**A.** The measurement station for the GMI phenomenon includes essential components: (1) Testing unit, (2) magnetic field generation Helmholtz coils, (3) Ammeter, (4) Compensating coils laboratory power supplies, (5) primary coil power supply, (6) PC, and (7) Electronic impedance measurement system and high-frequency LCR meter, **B**. Active GMI element: (1) reference resistor, (2) compensating coils, (3) amorphous tape, (4) analog-to-digital converter, (5) peak detector, and (6) differential amplifier. Graphical results are also presented in the image. Reproduced under the CC BY 4.0 license of MDPI [154].

The diverse experimental configurations and outcomes derived from MTJ sensors are presented in Fig. 7. The investigation shows that the MTJ signal response and local field potential (LFP) recordings exhibit similar forms, measured in millivolts (mV) and ohms (Ω). This was achieved using an in vitro setup with an MTJ sensor array to capture cerebral responses. Additionally, an in vivo method was employed to record neural responses from the rat cerebral cortex, utilizing a Wheatstone Bridge configuration with four TMR sensors, measuring neural response in picoteslas (pT). TMR devices, due to their compatibility with living organisms, promote neuronal growth, thereby advancing research in neurobiology and brain-computer interfaces [139]. Advancements in TMR technology offer innovative methods for pathogen identification, crucial for patient management and infection prevention. The fundamental principle involves the tunneling of electrons over the barrier, with the probability of tunneling being determined by the relative orientation of the magnetizations [28]. The minimal resistance is achieved through parallel magnetization, while the utmost resistance is achieved through antiparallel alignment. In numerous applications, the efficient operation of TMR sensors is contingent upon this association. The enhanced sensor-skin coupling of TMR sensors is a critical characteristic that is essential for the accurate collection of physiological data in wearable health monitoring devices. The precision and dependability of measurements are influenced by this connection [38]. To detect the subtle magnetic fields generated by biological processes, such as blood circulation and cardiac activity, TMR sensors are necessary [140,141]. To enhance adherence and measurement precision, TMR sensors may employ flexible substrates that imitate the skin contours to increase sensor-skin coupling. The sensor-skin contact is significantly influenced by the materials used in the production of TMR sensors, such as flexible ferromagnetic alloys such as NiFe and CoFeB [142,143]. The advancement of nanofabrication has led to the creation of TMR sensors that are more sensitive and compact [144,145]. Sensor efficacy can be influenced by skin attributes, including temperature, texture, and hydration [146]. To accommodate a wide range of epidermis conditions, researchers are conducting research on advanced signal processing systems. TMR sensors enhance accuracy by utilizing real-time calibration and adaptive algorithms to adjust measurements in response to the epidermis they encounter. Reliable physiological data from wearable health monitoring systems necessitates optimal sensor-skin contact [147]. Kurashima et al. [148]. Developed an MCG capable of accurately mapping heart magnetic fields at room temperature without requiring magnetically shielded rooms. This device offers higher spatial resolution compared to traditional electrocardiographs, enhancing the early diagnosis of ischemic heart disease and improving the assessment of ventricular arrhythmias. By enhancing the detectivity of TMR sensors to an average of 14.1 pTrms and employing a sensor array of 288 TMR sensors, they significantly reduced environmental noise using a mathematical algorithm for signal space separation. The resulting heart magnetic field resolution was measured at 0.99 pTrms, producing clear MCG comparable to those obtained with superconducting quantum interference device (SQUID) technology, thus positioning this MCG as a promising tool for cardiac health management. Tang et al. [149]. Introduced a novel TMR-based active electrode for ballistocardiography (BCG), aiming to enhance cardiac disease diagnostics, including sleep disorders and heart failure. Unlike conventional ECG, which requires contact sensing and ground electrodes, the TMR-based BCG sensor operates on a non-contact principle, eliminating the need for bulky equipment. Traditional BCG sensors often lack sensitivity and require extensive coverage of the chest area, limiting their use as wearables. The proposed active electrode consists of a TMR chip and an analog front-end (AFE) chip, which detects heartbeat-induced vibrations that cause geomagnetic changes. This signal is then amplified and digitized, offering a promising solution for improved BCG recording (see Fig. 8).

Rajas et al. [150]. Developed an eddy current circuit combined with a tunneling magnetoresistor (EC-TMR) to detect minute movements of orthopedic implants. They initially modeled a small three-turn rectangular eddy current loop (2.5 mm by 10 mm) using a full-wave electromagnetic simulator, followed by construction and testing with an analyzer. After optimizing the sensing element, the TMR sensor was fabricated and thoroughly characterized, achieving high-resolution micromotion detection at significant distances due to its high sensitivity and signal-to-noise ratio. However, the detection of micromotion indicated a risk of imminent failure. Siming et al. [151] presented a combined TMR array for nominal temperature magnetomyography (MMG) applications. Their TMR sensors, which exhibited an excellent signal-to-noise ratio, were capable of detecting MMG signals activated by electromyography (EMG) signals. They measured amplitudes of 200 pT and 30 pT corresponding to stressed and normal hand states, respectively, using finite element methods to account for the distance between the observation point and the magnetic field. Additionally, Huaming et al. [152] reported a prototype for contactless scanning that utilizes TMR sensors to quantify the presence of magnetic nanoparticles (MNPs) in lateral flow strips (LFSs). The sensing unit comprises two closely spaced parallel active TMR components, enabling perpendicular sensing relative to the magnetic field. The geometrical parameters of the setup were optimized based on the demagnetizing field produced by the test line. Future efforts will focus on making this biosensing platform more affordable and energy-efficient for portable applications.

In conclusion, TMR sensors signify important improvements in healthcare, especially with epidermal integration. The progress of equity in health surveillance technology requires accurate and uniform measurements across various skin tones. The effectiveness of TMR sensors is enhanced in numerous applications by employing advanced signal processing, flexible materials, and real-time calibration. TMR sensors are poised to enhance the accuracy of patient health status by early prediction that assure suitable estimations for all individuals, irrespective of their skin characteristics. Their versatility, sensitivity, and reliability are essential for delivering equitable healthcare, hence enhancing health outcomes for various communities.

### 3.5 Giant magnetoimpedance

GMI sensors are magnetic sensing instruments that use the giant magnetoimpedance phenomenon, characterized by a substantial change in the electrical impedance of certain conductive materials when subjected to an external magnetic field. This property facilitates the precise observation of fluctuations in the magnetic field [153].

**Fig. 9:**
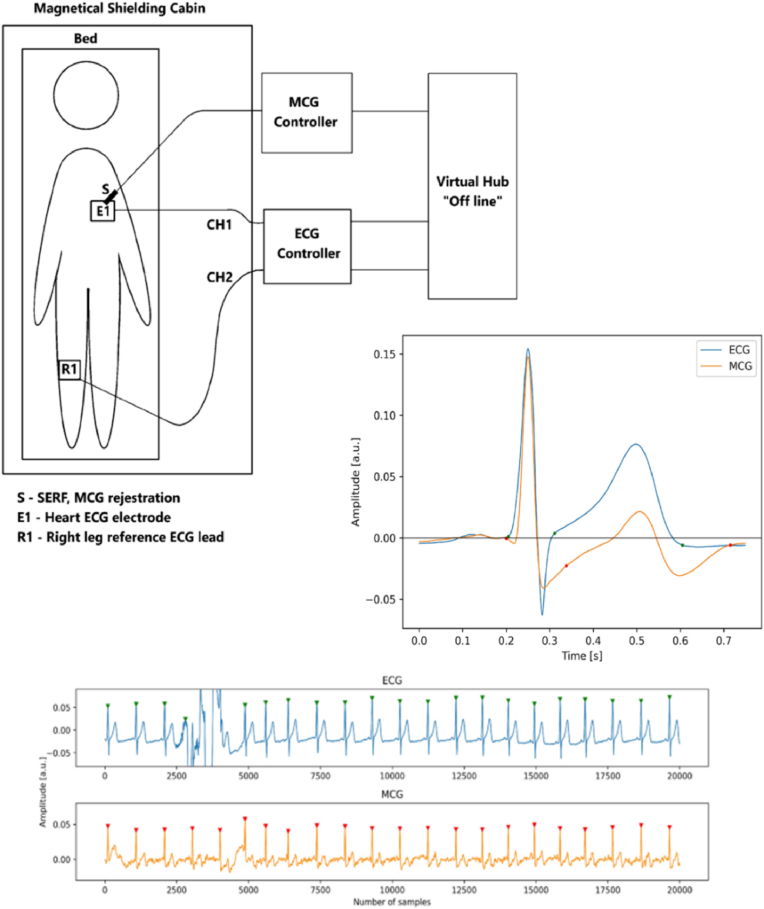
Measuring system for simultaneous ECG and MCG recording. Reproduced under the CC BY 4.0 license of Springer Nature [179].

**Fig. 10:**
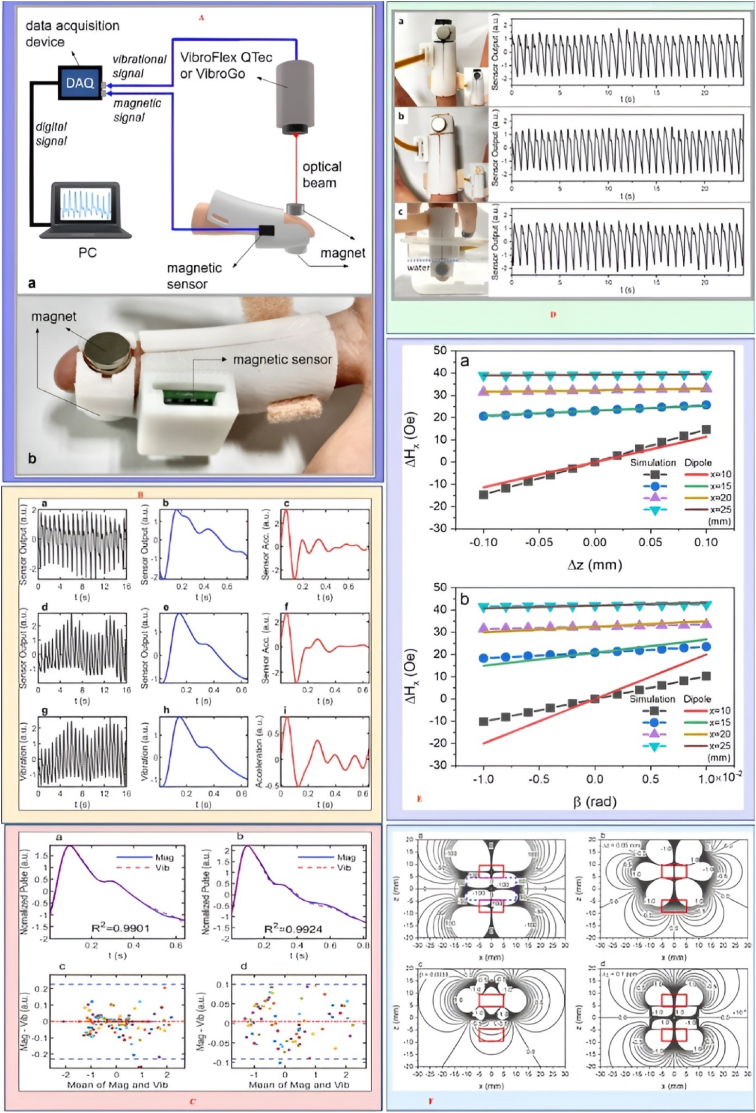
**A.** The measurement setup and performance of the fingertip-type MDVS are illustrated, including: (a) the measurement setup and (b) the performance metrics, **B.** Signals obtained from a subject include: (a) the magnetic sensor output signal over 16 s, (b) the average single pulse signal derived from (a), and (c) the second derivative of (b), (d)–(f) show the magnetic signals from another subject. For comparison, vibrational signals measured simultaneously include: (g) the time-dependent displacement signal, (h) the average single pulse signal, and (i) the average acceleration detected by the vibrometer. **C.** A comparison of single pulse magnetic and vibrational signals, with vibrational signals measured using VibroFlex QTec (a) and VibroGo (b). Bland–Altman plots based on repeated measurements using VibroFlex QTec (c) and VibroGo (d) are included, with blue dashed lines indicating ±1.96 SD and the red dash-dotted line representing the mean bias of differences between the two signals. **D.** Fingertip configurations: (a) wrapped in black tape, (b) wrapped in plaster, and (c) immersed in water, **E.** Contour plots depict: (a) Hx in the range of ±100 Oe, (b) △Hx due to a vertical shift of the top magnet by 50 μm, (c) △Hx from a pitch rotation of 0.0033 rad, and (d) △Hx induced by changes in blood susceptibility. The units for (a)–(c) are Oe, while (d) is in 10^−4^ Oe, with red boxes indicating the position of two cylindrical magnets and the blue dashed box in (a) showing the size and position of the finger cuboid. **F.** Comparison of field changes obtained through COMSOL simulation (symbols) and dipole approximation (solid line). Reproduced under CC BY 4.0 Springer Nature [180]. (For interpretation of the references to color in this figure legend, the reader is referred to the Web version of this article.)

A measurement station for studying the GMI phenomenon includes essential components such as Helmholtz coils, an ammeter, power supplies, and a high-frequency LCR meter shown in Fig. 9. A zoomed-in view highlights key parts of the GMI element, including a reference resistor and compensation coils [154]. GMI sensors are in biological applications, particularly in sensor-skin integration. Effective sensor-skin coupling is crucial for reliable physiological data, as skin contact largely affect the data quality and sensor performance, making it essential in wearable health monitoring systems [27]. Ferromagnetic materials are used in GMI sensors that show significant impedance fluctuations in alternating magnetic fields, allowing them to detect picoTesla-level magnetic fields. This sensitivity is invaluable for monitoring critical indicators like electrical impulses in the brain and heart [37]. Skin type, moisture content, and surface texture affect sensor adhesion and efficacy. Flexible substrates in GMI sensors can adapt to skin contours, improving signal detection and enhancing patient comfort and monitoring accuracy. Soft magnetic materials, such as amorphous alloys of Co or Fe, are used for magnetic field sensing. A magnetic scanning microscope built around an offdiagonal magneto-impedance sensor has been introduced by Gudoshnikov et al. [155]. In their system, a pick-up coil with 70 turns was wound around a 4 mm segment of glass-covered microwire sensor with 13.5 m metallic core diameter constructed of CoFeCrSiB. Due to the orientation of the GMI sensor, there is a separation of around 200 m between the sample and the tip of the microwire. A positioner was used to move the sample and the GMI sensor. It generates flimsy signals that depend on the temperature. Wang et al. [156] explored the use of magnetic sensors across various applications, including navigation, power distribution, robotics, factory automation, and medical diagnostics. They achieved a giant magneto-impedance of 41,036 % in commercially available ferrite core inductors. By directly measuring the impedance of these inductors without optimization, they attained a magnetic field detection limit of 10 nT at 1 Hz. When a 100-pF capacitor was connected in series with the inductor, the detection limit improved to 625 pT at 1 Hz within a series RLC resonator configuration. This enhancement can be attributed to the magnetic field-dependent self-resonance of the inductors, which function as lumped RLC resonators. In comparison to traditional electromagnetic induction sensing methods, the magneto-impedance sensing approach demonstrated a remarkable 5000-fold improvement in magnetic field detection capability (see Fig. 10).

**Fig. 11:**
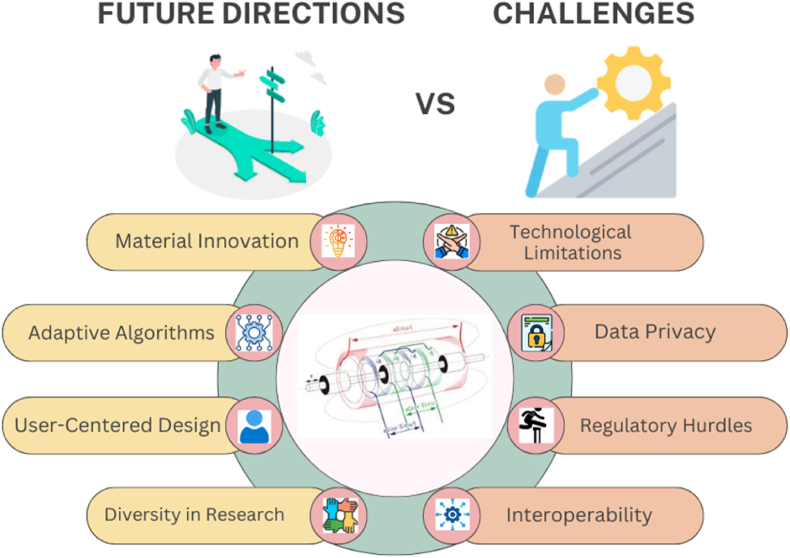
Visualizing the path forward: Innovations and limitations in magnetic sensors.

Gazda et al. [157] introduced a novel GMI sensor based on a compensation measurement principle, utilizing the local ‘zero-field’ minimum of its double-peak characteristic as a sensitive null detector. A compensation field was applied in real-time using a microprocessor-based, two-step quasi-Newtonian optimization method. The paper describes the optimization of material parameters through Joule-annealing of selected amorphous alloys. Test results from the prototype demonstrate a linear output characteristic, low measurement uncertainty, and resilience against time and temperature drift. Skin characteristics like humidity and surface roughness influence sensor performance. To monitor these variations advanced signal processing algorithms are developed. Real-time calibration and adaptive algorithms can enhance measurement accuracy by adjusting sensor skin coupling [158]. GMI sensors enable continuous health monitoring, assessing major signs such as respiratory patterns, heart rate, and muscular activity, crucial for medical professionals. Their reliable performance across various skin tones ensures precise findings for various populations, thereby enhancing healthcare equity by reducing the risk of misdiagnosis. The application of adhesives to affix wearable GMI sensors to the skin enhances the sensor skin coupling effect, hence facilitating more precise monitoring of physiological signals. The deployment of these sensors in areas rich in physiological activity guarantees the acquisition of reliable data over a prolonged duration. GMI sensors identify biological markers linked to current health conditions by utilize magnetic nanoparticles. This facilitates enhanced disease management and prompt diagnosis. They can be integrated into a diverse array of medical equipment, including those utilized for minimally invasive procedures, offering interventions that are accurate and anatomically aligned. The interaction between GMI sensors and the skin significantly influences the efficacy of these sensors in biological applications, as previously mentioned. The reliability and effectiveness of these sensors for monitoring physiological signals can be enhanced by innovations in material properties, design, and signal processing technologies. GMI sensors must exhibit precision and reliability across diverse skin types to effectively deliver equitable healthcare solutions [159].

Recent advancements in scientific knowledge and technology capabilities position GMI sensors to transform health monitoring and diagnosis. These sensors will deliver assessments to patients that are rapid, precise, and customized to their individual requirements. This evolution will precipitate a paradigm shift in personalized care, resulting in enhanced patient outcomes and increased accessibility to health monitoring.

## 4 Advanced signal processing techniques

### 4.1 Noise reduction and signal enhancement

Advanced signal processing methods are crucial for enhancing the accuracy of sensor readings, particularly in biomedical applications where precision is vital. One of the primary challenges in obtaining reliable data from sensors is the noise introduced by variations in the epidermis, which can significantly affect measurement outcomes [160]. To address this challenge, various noise reduction techniques are employed. These methods involve sophisticated algorithms designed to filter out unwanted signals while preserving the integrity of the desired data. For instance, real-time sensor sensitivity algorithms can adapt the sensor's response based on the specific characteristics of the skin it is in contact with. This adaptability improves the accessibility and accuracy of measurements across diverse skin conditions, ensuring that sensors perform optimally regardless of the user's unique biological characteristics [161]. In addition to real-time adjustments, optimization techniques play a vital role in enhancing signal quality. By systematically reducing noise levels, these techniques enhance the quality of data collected, which is essential for accurate feature extraction. This process involves analyzing the signal to identify and remove artifacts that do not contribute to the underlying physiological parameters being measured [162]. The implementation of these advanced signal processing methods not only improves the reliability of sensor data but also increases the overall effectiveness of biomedical devices. As the demand for precise monitoring solutions grows, these enhancements are crucial for the development of next-generation healthcare technologies that can operate seamlessly across a wide range of user demographics and conditions.

### 4.2 Machine learning applications

Machine learning algorithms have become increasingly important in enhancing the predictive accuracy of biomedical measurements. These algorithms enable the analysis of complex datasets, allowing for more precise interpretations of physiological signals [163]. A notable example of this application is the work by Ahmed S. Alghamdi et al. [164], who developed a method for measuring and predicting blood pressure using oscillometric waveforms. In their approach, the researchers segmented the oscillometric waveform into three distinct periods: the initial phase leading up to the SBP, the interval between SBP and DBP, and the final phase from DBP to the end of the waveform. This careful segmentation is crucial because each period contains unique information that can influence blood pressure readings. By training classifiers on a dataset with labeled attributes corresponding to systolic, diastolic, and other cardiac beats, they were able to employ several machine learning algorithms specifically k-nearest neighbor (kNN), weighted k-nearest neighbor (WkNN), and Bagged Trees. These classifiers effectively estimated SBP and DBP, demonstrating the potential of machine learning to enhance diagnostic capabilities in cardiovascular monitoring. Another significant advancement in this field is presented by Eom et al. [165], who introduced an innovative end-to-end deep learning architecture specifically designed for continuous blood pressure monitoring. Their model utilizes raw physiological signals from multiple sources, including ECG, ballistocardiogram, and PPG, without the need for traditional feature extraction. This is a notable advancement, as feature extraction often requires domain-specific knowledge and can introduce biases or errors. Eom et al.'s architecture integrates a convolutional neural network (CNN) with a bidirectional gated recurrent unit (BiGRU), combining the strengths of both deep learning techniques. The CNN effectively captures spatial features from the input signals, while the BiGRU processes temporal dependencies, allowing the model to learn patterns over time. Their results showed that this integrated approach significantly outperformed conventional methods, highlighting the power of deep learning in real-time health monitoring applications.

Overall, these machine learning applications underscore the transformative potential of advanced algorithms in improving the accuracy and reliability of physiological measurements, paving the way for more effective health monitoring solutions.

### 4.3 Hardware innovations

In healthcare technologies, hardware innovations play a critical role in advancing the capabilities of diagnostic and signal processing systems. Azghadi et al. [166] conducted an in-depth exploration of deep learning accelerators and neuromorphic processors, focusing on their applications in edge computing for healthcare. This approach is particularly significant as it allows for data processing to occur closer to the source, thereby reducing latency and improving the responsiveness of medical devices. The authors emphasize the use of advanced technologies such as memristive devices and Field Programmable Gate Arrays (FPGAs). Memristive devices, known for their ability to mimic synaptic functions in the human brain, are particularly promising for applications requiring adaptive learning and memory retention. These devices can enable more efficient processing of complex biomedical signals, making them ideal for tasks that require real-time analysis and decision-making. FPGAs, on the other hand, offer a flexible and reconfigurable platform for implementing deep learning algorithms. Their parallel processing capabilities allow for rapid computation, which is essential for handling large datasets generated by medical IoT systems and Point of Care (PoC) devices. By leveraging FPGAs, healthcare applications can achieve higher throughput and lower power consumption compared to traditional processing units.

### 4.4 Continuous monitoring techniques

Continuous monitoring of physiological parameters, such as blood pressure, is essential in modern healthcare due to its ability to provide real-time insights into a patient's condition [2]. This approach is particularly important for managing chronic diseases, detecting acute changes in health status, and improving overall patient outcomes [23]. Continuous monitoring allows healthcare providers to make timely interventions, track the effectiveness of treatments, and personalize care based on individual patient responses. As a result, the demand for reliable and accurate continuous monitoring systems has surged, leading to innovations in sensor technologies and data analysis methods. Ebrahim et al. [56] investigated a novel approach to continuous blood pressure measurement by integrating technologies such as continuous wave radar (CWR), photoplethysmogram (PPG), and electrocardiogram (ECG) sensors. Their research aims to address the challenges associated with conventional blood pressure measurement techniques, which often rely on intermittent readings that can miss critical fluctuations in a patient's condition. In their study, Ebrahim et al. employed wavelet transform and adaptive filtering techniques to effectively reduce noise from the signals captured by these sensors. The wavelet transform is particularly beneficial for analyzing non-stationary signals, such as those produced by physiological processes, as it allows for multi-resolution analysis. This means that both high-frequency noise and low-frequency trends can be addressed simultaneously, leading to cleaner and more accurate signal representations. Adaptive filtering further enhances the accuracy of the measurements by dynamically adjusting the filter characteristics based on the incoming signal characteristics. This adaptability ensures that the system can respond to varying noise conditions and different patient states, which is crucial during activities such as exercise or changes in posture.

### 4.5 Cardiovascular disease detection

To address the growing issue of CVD, researchers have been developing innovative diagnostic tools that leverage advancements in technology and machine learning. For instance, Su et al. [167] and Phua et al. [168] created a portable electrocardiogram (ECG) acquisition system that utilizes machine learning algorithms to accurately detect potential cardiac abnormalities. This system is designed for ease of use, allowing patients to monitor their heart health conveniently at home or in remote settings. By employing a combination of traditional statistical methods and advanced deep learning models, the system achieved an impressive classification accuracy of 99.13 %. This high level of accuracy is crucial for ensuring that potential cardiovascular issues are identified early, enabling timely medical intervention. Similarly, Kolhar et al. [169] highlighted the importance of automated ECG analysis in improving the efficiency and accuracy of cardiovascular diagnostics. Their research employed artificial intelligence (AI) models to analyze ECG data, achieving test accuracies of 99 %. Such automation not only helps in reducing the workload of healthcare professionals but also minimizes the risk of human error, ensuring that patients receive accurate assessments of their heart health. In addition to ECG-based approaches, Huang et al. [170] explored the utility of magnetocardiogram (MCG) parameters for detecting coronary artery disease (CAD). MCG is a non-invasive technique that measures the magnetic fields produced by the electrical activity of the heart. By utilizing multilayer perceptron neural network models, Huang et al. achieved detection accuracies ranging from 71.2 % to 90.5 %. This research underscores the potential of MCG as a valuable tool in clinical diagnostics, particularly for patients who may not respond well to traditional ECG methods. Overall, these studies demonstrate the significant advancements in cardiovascular disease detection through the integration of machine learning and novel diagnostic technologies. By enhancing the accuracy and efficiency of cardiovascular assessments, these innovations hold promise for improving patient care and outcomes in the face of rising cardiovascular disease prevalence.

### 4.6 Material use in sensors

The effectiveness and reliability of sensors in biomedical applications heavily depend on the materials used in their construction [171].

#### 4.6.1 Hall Effect Sensors (HESs)

• HESs primarily use semiconductor materials such as silicon and gallium arsenide (GaAs). These materials exhibit significant Hall voltage when exposed to magnetic fields, making them ideal for applications in magnetic field sensing and positioning [172].

#### 4.6.2 Anisotropic Magnetoresistance (AMRs)

• AMRs typically utilize ferromagnetic materials like nickel, iron, and cobalt. These materials exhibit a change in electrical resistance depending on the angle of the magnetic field relative to the current flow, enabling precise magnetic field measurements [173].

#### 4.6.3 Giant Magnetoresistance (GMR)

• GMR sensors are often constructed using multilayer thin films composed of alternating ferromagnetic and non-magnetic materials, such as iron and copper. The unique structure allows for a dramatic change in resistance in response to external magnetic fields, making GMR sensors highly sensitive and suitable for data storage and sensing applications [174].

#### 4.6.4 Tunnel Magnetoresistance (TMR)

• TMR sensors are based on magnetic tunnel junctions, which consist of two ferromagnetic layers separated by a thin insulating barrier, often made from aluminum oxide (Al2O3). The tunneling effect in this structure provides a significant change in resistance when subjected to a magnetic field, enhancing sensitivity [175].

#### 4.6.5 Giant magnetoimpedance (GMI)

• GMI sensors utilize amorphous magnetic materials, such as CoFeSiB alloys. These materials exhibit a substantial change in impedance in response to varying magnetic fields, making them useful for magnetic field sensing in various applications [176].

In summary, the choice of materials in these magnetoresistive sensors is critical for optimizing their sensitivity, performance, and application in various fields, including medical diagnostics, automotive, and data storage technologies [177,178].

The fingertip-type magnetically detected vibration sensor (MDVS) was tested under various conditions, including tape-wrapped, plaster-wrapped, and water-immersed fingertips, demonstrating robustness [180].

## 5 Challenges and limitations

Despite advancements, the sensor-skin coupling effect continues to be impeded by obstacles and constraints. The technological constraints of magnetic sensor technologies present significant challenges [181,182]. It is a difficult task to reduce skin characteristics while simultaneously improving sensitivity and precision [183]. The calibration procedures are resource-intensive, which makes it difficult to implement across a wide range of industrial sizes [184]. The production costs may be inflated using novel materials and complex calibrating methods, which may raise concerns about accessibility for disadvantaged populations [185]. The adoption of technology is contingent upon user acceptability. Individuals may refrain from employing magnetic health monitoring devices if sensor-skin coupling issues undermine their reliability. To achieve success, these technologies must be reliable and effective for a wide range of demographics [186]. Health monitoring technology can be enhanced and equitable healthcare for all individuals can be promoted by investigating sensor interactions with diverse skin attributes. Tackling issues of sensor-skin coupling would enhance patient outcomes and reinforce confidence in healthcare technologies [187,188]. A comparative analysis of diverse magnetic sensor technologies is presented in Table 3, focusing on essential performance metrics such as sensitivity, power consumption, temperature stability, angular detection capabilities, linear range, and detectivity. This analysis highlights the strengths and weaknesses of each technology.

**Table 3:** Comparison of magnetic sensor technologies based on key performance parameters.

Technologies				
Parameters	**TMR**	**Hall Effect**	**GMR**	**AMR**
High Sensitivity	High	Moderate	High	Low
Low Power Consumption	Low	Moderate	Moderate	High
Temperature Stability	High	Low	Moderate	Low
Angle Detection	360°	360°	Limited	Limited
Linear Range (Oe)	∼1000	>10,000	∼100	∼10
Detectivity (nT/Hz^1/2)	∼0.1	>100	∼10	∼1
Applications	Magnetic field sensing	Current, position sensing	Magnetic field sensing	Magnetic field sensing
Health Status Detection	Suitable	Suitable	Suitable	Suitable

## 6 Discussion and magnetic sensors equity

This literature review on magnetic sensors highlights its transformative potential and significant obstacles in the domain of biological healthcare. Skin coupling effects have been noted, potentially diminishing measurement accuracy. HES, AMR, TMR, and GMI magnetic sensors are being utilized in diagnostic instruments and wearable health devices. The characteristics of skin, including pigmentation, hydration, and texture, can significantly affect the effectiveness of these products. This variation prompts apprehensions regarding the dependability of health evaluations for underrepresented populations. The sensor-skin coupling problem highlights the challenges of physiological monitoring, making it critically important. Variations in skin features that lead to discrepancies in sensor readings affect patient faith in health technology and therapeutic effects. This research indicates that some difficulties remain, despite recent developments in sensor technology, including adaptive calibration methods and enhanced materials. To influence healthcare equity, these technologies must be both effective and accessible to diverse populations.

### 6.1 Practical considerations: life cycle, economics, and market trends

While the technical performance of magnetic sensors is critical for biomedical applications, their real-world adoption hinges on practical factors such as environmental impact, cost-effectiveness, and market readiness. Here, we evaluate these dimensions to provide a holistic perspective on the translational potential of magnetic sensor technologies.

#### 6.1.1 Life cycle assessment (LCA)

LCA is a comprehensive evaluation of the inputs, outputs, and potential environmental impacts of a product throughout its entire life cycle [189]. According to ISO 14040/44, LCA consists of four key stages: (1) goal and scope definition, (2) life cycle inventory analysis, (3) life cycle impact assessment (LCIA), and (4) interpretation of results.

The goal and scope definition are the most critical step in an LCA, as it establishes the precise utility of the product and outlines the product system, including assumptions, allocations, and system boundaries. The life cycle inventory (LCI) involves collecting quantified input and output data such as mass and energy metrics throughout the product's life cycle, from raw material extraction and production to application and eventual waste treatment or recycling.

During the LCIA phase, potential environmental impacts are assessed based on the LCI data. Before this assessment, it is essential to identify relevant environmental categories, indicators, and methods for the characterization model [190].

Magnetic sensors provide compelling sustainability benefits compared to optical sensors like PPG, primarily due to their durable construction, reduced consumable requirements, and superior energy efficiency. The inherent design of magnetoresistive sensors (including HES, AMR, GMR, TMR, and GMI variants) incorporates long-lasting thin-film alloys that enable repeated use, significantly decreasing electronic waste generation in clinical environments by up to 60 % relative to disposable optical electrodes. Their passive operation through magnetic field measurements eliminates the need for constant light emission, resulting in 40–60 % lower power consumption that substantially extends battery life in wearable applications. While these sensors do contain rare-earth materials such as neodymium, recent advances in urban mining techniques demonstrate the potential to recover over 75 % of these valuable components, though scaling these recycling processes to industrial levels remains an ongoing challenge. These combined advantages in material longevity, energy conservation, and end-of-life recovery position magnetic sensor technology as an environmentally sustainable solution for next-generation medical monitoring systems [191].

#### 6.1.2 Economic viability

The economic viability of magnetic sensors has significantly improved through recent advancements in fabrication and material science [192]. Magnetic sensors inherent resistance to motion artifacts and environmental interference substantially decreases recalibration requirements, resulting in lower long-term maintenance costs for healthcare deployments [193]. The integration of these sensors with CMOS technology has additionally enabled mass production scalability, with industry projections indicating a potential 30 % cost reduction for implantable magnetic sensor systems by 2030, making them increasingly attractive for widespread medical adoption [192].

#### 6.1.3 Marketability and adoption barriers

The market landscape for magnetic health sensors presents both significant growth opportunities and notable adoption challenges, with the global market projected to expand at a compound annual growth rate of 8.2 % from 2025 to 2030, fueled primarily by increasing demand for non-invasive monitoring solutions among aging populations. Consumer wearable devices incorporating advanced technologies like magnetic plethysmography exemplified by smart rings that have recently obtained FDA clearance for hypertension monitoring are successfully bridging the gap between clinical and home healthcare applications. However, market penetration faces substantial barriers including stringent biocompatibility requirements under ISO 10993 standards and protracted regulatory approval timelines, despite recent progress through modular sensor designs that streamline certification processes. Persistent cost disparities continue to limit accessibility, prompting innovative solutions such as subsidized deployment initiatives in low-resource settings through industry consortia, which aim to address healthcare inequities while expanding market reach. These competing dynamics of technological advancement versus implementation challenges will likely shape the sector's development trajectory in coming years [[194], [195], [196]].

## 7 Future directions

The effectiveness and availability of magnetic sensor technology can be significantly enhanced by focusing on several key areas:

***Material Innovation:*** Advancements in biocompatible and flexible materials, such as elastomers and conductive polymers, are essential for improving sensor performance. These materials can mitigate the effects of varying epidermal qualities on readings, enhancing both user comfort and data accuracy. For instance, integrating materials like polyimide or silicone-based substrates could improve conformability and durability, leading to more reliable sensor outputs.

***Artificial Intelligence and Machine Learning:*** The integration of AI and machine learning algorithms can transform calibration methods for magnetic sensors. By employing adaptive calibration techniques, sensors can adjust their readings based on individual skin characteristics, such as hydration levels and texture. For example, using machine learning models trained on diverse skin types can enhance measurement precision by compensating for variations in sensor-skin interactions.

***User-Centered Design:*** A robust user-centered design approach in wearable health devices is critical. This involves conducting iterative usability testing with diverse demographic groups to gather feedback on comfort, functionality, and aesthetics. Incorporating features such as adjustable fit and customizable interfaces can significantly improve user acceptance and satisfaction.

***Accessibility and Affordability:*** Addressing the accessibility of magnetic sensor technologies is crucial. Collaborative efforts among industry stakeholders, healthcare providers, and government entities should focus on developing low-cost solutions. For instance, leveraging open-source designs and scalable manufacturing techniques can help create affordable sensors for underserved communities, preventing technological advancements from widening health disparities.

***Longitudinal Studies:*** Conducting longitudinal studies to evaluate the long-term functionality of magnetic sensors across various epidermal types is vital. These studies should focus on quantifying sensor drift, reliability, and user adherence over extended periods. Such research will provide critical insights into optimal operational periods and facilitate more accurate health assessments.

***Diversity in Research:*** Future investigations should prioritize inclusive participant recruitment to ensure findings are applicable to a broader population. This includes enrolling individuals across different age groups, skin tones, and health conditions to enhance the relevance of research outcomes. A diverse participant pool will support the development of universally effective magnetic sensor technologies.

Collectively, these directions provide a roadmap for researchers and developers to enhance sensor performance, increase accessibility, and ensure the technology meets the diverse needs of users.

## 8 Conclusion

In conclusion, magnetic sensors have the potential to revolutionize biomedical healthcare through non-invasive monitoring, thanks to their adaptability. However, skin coupling effects present significant challenges that must be addressed to ensure equitable health assessments across diverse populations. Emphasizing novel materials, adaptive technologies, user-centric designs, and inclusive research is crucial for enhancing the reliability and accuracy of magnetic sensors.

Moreover, understanding the life cycle assessment and economic viability of these technologies is essential for promoting sustainability and marketability. As we advance technologically, it is imperative to rectify health disparities to foster an equitable healthcare system. Future research and collaboration will not only improve magnetic sensor technologies but also enhance health outcomes, creating a more inclusive healthcare environment for all demographics. These initiatives will contribute to equitable health monitoring systems, ultimately improving healthcare outcomes for everyone, regardless of skin type.

## CRediT authorship contribution statement

**Wasim Ullah Khan:** Writing – review & editing, Writing – original draft, Visualization, Validation, Methodology, Investigation, Formal analysis, Conceptualization. **Mohammed Alissa:** Writing – review & editing, Visualization, Methodology, Formal analysis. **Huawei Ma:** Visualization, Validation, Supervision, Conceptualization. **Uzair Aslam Bhatti:** Writing – original draft, Visualization, Validation, Conceptualization. **Abdullah Alghamdi:** Methodology, Data curation, Conceptualization. **Mohammed A. Alshehri:** Writing – review & editing, Visualization, Investigation. **Abdullah Albelasi:** Writing – review & editing, Validation, Investigation, Conceptualization.

## Declaration of competing interest

The authors declare no conflict of interest.

## Data Availability

No data was used for the research described in the article.
